# Rubisco dark inhibition in angiosperms shows a complex distribution pattern

**DOI:** 10.1093/jxb/erag090

**Published:** 2026-04-15

**Authors:** Connor Nehls-Ramos, Elizabete Carmo-Silva, Douglas J Orr

**Affiliations:** Lancaster Environment Centre, Lancaster University, Lancaster LA1 4YQ, UK; Lancaster Environment Centre, Lancaster University, Lancaster LA1 4YQ, UK; Lancaster Environment Centre, Lancaster University, Lancaster LA1 4YQ, UK; Western Sydney University, Australia

**Keywords:** CA1P, chloroplast, dark inhibition, enzyme activity, inhibitors, photosynthesis, photosynthetic metabolism, Rubisco, sugar-phosphates

## Abstract

Crop yields can be improved through making photosynthesis more efficient. The regulation of the CO_2_-fixing enzyme Rubisco during fluctuating light conditions limits productivity and is a target for improvement. Regulation in low light and darkness by accumulation of Rubisco inhibitors, predominantly via 2-carboxy-D-arabinitol 1-phosphate, has been known for over four decades but an explanation is still lacking for its physiological role and high variability across species. We compiled all published data for dark inhibition of Rubisco in flowering plants and investigated phylogenetic trends. Literature data for 157 species across 14 orders was compared and standardized, categorized into four dark inhibition levels, and analysed in the context of current phylogenetic information. We created a novel resource for Rubisco dark inhibition across flowering plants, highlighting clear gaps and biases in the available data, while also raising further questions on the evolution of this trait. Our work supports better understanding of the enigmatic process of photosynthetic regulation by Rubisco dark and low light inhibition and informs future efforts in enhancing photosynthesis in crops.

## Teaser

Available data on Rubisco dark inhibition show a complex distribution of levels of its inhibition in flowering plants. This suggests that there may be chloroplast microenvironment drivers for low versus high Rubisco inhibition.

## Introduction

Increasing agricultural productivity and resilience can contribute to increasing the sustainability of crop yields in the face of climate change (Li *et al*., 2025). One strategy to improve crop output is by improving photosynthesis through fixing inefficiencies associated with Rubisco, the imperfect enzyme essential to carbon fixation in photosynthesis (Croce *et al*., 2024). Despite advances in understanding the complex regulation of Rubisco activity, there remain many large knowledge gaps (Parry *et al*., 2008; Leister *et al*., 2023; Orr *et al*., 2023; Pasch *et al*., 2024). Passing clouds, wind, and sunflecks result in irregularities in lighting throughout the crop canopy. For many but not all crops, accumulation of Rubisco inhibitors has been reported when leaves experience an extended low light or dark period.

The identification of dark and low light Rubisco inhibition arose first due to anomalies in measuring the activation state of Rubisco in leaf extracts (Vu *et al*., 1984). Total activity measurements—incubating leaf extracts in an activating buffer containing Mg^2+^ and CO_2_—showed a stark depression in activity in pre-dawn samples compared with midday leaves in certain species. The *in vitro* carbamylation of Rubisco enhanced binding of dark- and low-light-accumulated inhibitory compounds present in the leaf extracts, a process generally referred to as dark inhibition (Servaites, 1985; Holbrook *et al*., 1992). The accumulation of inhibitors in the chloroplast is a slow process requiring hours in full darkness to reach peak inhibitor levels (Andralojc *et al*., 1994). In species with particularly high dark inhibition of Rubisco, for example French bean (*Phaseolus vulgaris*) and potato (*Solanum tuberosum*), 2-carboxy-D-arabinitol 1-phosphate (CA1P) could explain the majority of activity loss in the dark (Seemann *et al*., 1985; Gutteridge *et al*., 1986; Berry *et al*., 1987). Interestingly, as more species were studied for dark inhibition, it became clearer that some plants lacked any appreciable accumulation of dark inhibitors (Holbrook *et al*., 1992).

Dark inhibition varies greatly between flowering plant species and, to a lesser extent, within varieties of the same species (Servaites *et al*., 1986; Holbrook *et al*., 1992, 1994; Sage, 1993; Orr *et al*., 2023). While the pathway for the *in vivo* synthesis of CA1P has yet to be conclusively resolved, the precursor molecule 2-carboxyarabinitol is suggested to be phosphorylated by a kinase with activity stimulated in low light (Andralojc *et al*., 1996a, b, 2002; Parry *et al*., 2008). Regularly utilizing a kinase is resource and energy costly, implying an advantage to the accumulation of CA1P in the dark and low light; that is, this inhibitor may provide benefits for plants depending on various environmental and metabolic pressures. Alternatively, CA1P accumulation may be a byproduct of an as yet unknown metabolic pathway not harmful enough to be selected out by certain environments. Various hypotheses exist for the utility of dark inhibition or CA1P accumulation, but no conclusive *in vivo* evidence has yet been provided (Andralojc *et al*., 1996a; Khan *et al*., 1999; Van Heerden *et al*., 2003; Parry *et al*., 2013). The high variability in levels of accumulation, even across closely related species, suggests further nuance in the evolution and potentially the function of this trait.

While previous works have discussed trends in dark inhibition by phylogeny, no comprehensive list has been made across flowering plants (Holbrook *et al*., 1992; Sage, 1993; Orr *et al*., 2023). To better understand the natural variation in dark inhibition, we have compiled the averaged species dark inhibition data from the available literature for 157 species across 14 orders and organized these data by phylogeny and levels of dark inhibition. Data were also analysed and filtered by experimental methods, to ensure that the data used were comparable, and then summarized at species levels. Trends in dark inhibition level by phylogeny, photosynthetic metabolism type, and gaps in dark inhibition data from species to order level suggest distinct order- to species-level evolutionary patterns in flowering plants and that dark inhibition may be older than flowering plants.

## Materials and methods

### Dark inhibition database

Dark inhibition data were collected from an extensive search of the literature published, as of August 2025, using a combination of Google Scholar (https://scholar.google.com/schhp?hl=en&as_sdt=0,5), Web of Science (https://www.webofscience.com/wos/woscc/basic-search), Scopus (https://www.scopus.com/pages/home?display=basic&zone=header&origin=#basic), and a further search in the references of all papers found that contained information pertaining to Rubisco activity in light- and dark-adapted leaf samples. The keywords used for these searches were: ‘Rubisco’, ‘Dark’, ‘Inhibition’, ‘Diurnal’, ‘Total Activity’, ‘CA1P’, and ‘2-carboxy-D-arabinitol 1-phosphate’. These keywords were used in various combinations to maximize paper identification. The focus of these analyses is within flowering plants and so three species outside this criterion were not included for further analysis. In total, 45 sources were found with 331 data points for dark inhibition. Each datapoint is representative of a given genotype sampled within each given study, or of the same genotype at different stages of growth. The full dataset is within the GitHub repository https://github.com/cwnehls/NehlsRamos_etal_JxB_BriefComms_Code_and_Dataset in the excel file NehlsRamos_etal_JxB_BriefComms_Data.

The dark inhibition ratio was calculated as [1−(dark-adapted Rubisco total activity/light-adapted Rubisco total activity)]. For dark inhibition levels, this ratio is multiplied by 100 to represent the percentage of dark inhibition. Total Rubisco activity is the *in vitro* carboxylation activity for fully carbamylated Rubisco from leaf samples. For sources that did not include total activity values but had total activity plotted, the data were estimated using SplineCloud plot digitizer (https://splinecloud.com/). Further details on light levels, literature sources, and more are contained in the dark inhibition dataset excel file.

Species phylogenetic information was collected using the angiosperm phylogeny website, NCBI taxonomy browser, The Kew Tree of Life, and the most recent literature on plant taxonomic groupings (Ruggiero *et al*., 2015; Baker *et al*., 2022; Sayers *et al*., 2025; Stevens, 2025).

Data quality assessment for final analysis included preliminary comparison of variation at species level of the three main experimental methods used for activity determination in the literature collected, 3-phosphoglyceric acid (3PGA)-NADH-coupled assays, radiolabelled CA1P assays, and radiolabelled CO_2_ assays (Supplementary Fig. S1A). Results were inconsistent for the first two assay methods for species dark inhibition values, compared with the radiolabelled CO_2_ method. While the 3PGA-NADH method did not have significant differences for the proportion of dark inhibition between dark- and light-treated samples, such as in wheat (Sales *et al*., 2020), the lack of data with these methods means that there is insufficient information to determine more conclusively if the results from these methods are comparable with the radiolabelled method that has been used for the large majority of species. To minimize unnecessary unknown variation while retaining the highest amount of quality data, only the radiolabelled CO_2_ method was kept for further analysis.

### Radiolabelled method data validation

Analysis was performed for the difference in reagent concentration, assay timing, plant sampling conditions, as well as pH and temperature of assays. This did not identify significant trends by linear regression, meta-regression, and Kruskal–Wallis tests, besides the light level for dark treatments and EDTA concentrations. Slight differences in methods, such as addition of MgCl_2_ in the sample extraction buffer (Sharwood *et al*., 2016) or purity of ribulose 1,5-bisphosphate (RuBP) (Andralojc et al., 2012), can have significant effects on recorded total Rubisco activity. However, in the context of the proportion of activity from light- and dark-treated samples, these discrepancies did not provide significant statistically identifiable differences that could otherwise be explained by species-level variation (Supplementary Fig. S1B; see code). Furthermore, at the species level for *P. vulgaris*, the most sampled species, a Kruskal–Wallis test detected a significant but relatively small difference with light level of sampling. In *Glycine max*, the next most sampled species, this difference was not found. It was concluded that there was insufficient evidence for variation within the radiolabelled CO_2_ method applications that would justify removal of specific studies, and so all data points for flowering plants using these methods were kept for further analysis. The final count for literature sources was 39, with 312 data points on dark inhibition, 157 of which are from unique species (see full dataset).

### Dark inhibition levels

By comparison of normal distribution, Gaussian fit, and data point clustering, four main categories of dark inhibition were identified within the current data. The distribution of the data at the order level was determined to be not normal by Hartigan’s dip test. To visualize if there was clustering of dark inhibition values, a combination of Gaussian mixture models by McLust, Silverman’s tests for modes, and kernel density estimate modelling showed four distinct peaks within the distribution of species dark inhibition values (Supplementary Fig. S2A). The antimodes, or valleys, between these modal peaks remained similar with and without the presence of the Fabales data, which was tested to ensure that over-representation of Fabales did not skew the categorizations (Supplementary Fig. S2B, C). These levels of dark inhibition are described as low (<18%), moderate (18–44%), high (44–77%), and very high (>77%) (Supplementary Fig. S2A). While high variation of dark inhibition values was found even at the species level for some species (Table 1), the presence of the four modes provided a range of values that could be used to describe the general distribution of dark inhibition at species, genus, or order level for identification of further trends.

**Table 1:** Summary of Known Species Dark Inhibition levels by Order and Tribe

Order	Tribe	Species	% Mean Dark Inhibition
Nym	*N/A*	*Nuphar lutea*	**84(VH)**
Ali	*N/A*	*Hydrilla verticillata^a^* ^*c*^	−7.0(L)
Ali	*Colocasieae*	*Alocasia macrorrhiza*	*39*±*0(M)*
Asp	*Vandeae*	*Arachnis x Ascocentrum x Vanda*	17(L)
Poa	*N/A*	*Bromelia pinguin* ^*c*^	**96(VH)**
Poa	*N/A*	*Ananas comosus* ^*c*^	**97(VH)**
Poa	*Cypereae*	*Cyperus esculentus^a^*	−5.0(L)
Poa	*Cypereae*	*Cyperus iria^a^*	11(L)
Poa	*Oryzeae*	*Oryza sativa*	48±18(H)
Poa	*Triticeae*	*Hordeum vulgare*	3.0(L)
Poa	*Triticeae*	*Triticum aestivum*	−1.0±2.0(L)
Poa	*Zoysieae*	*Zoysia japonica^a^*	*27*±*1.0(M)*
Poa	*Cynodonteae*	*Cynodon dactylon^a^*	*1.0*±*10(L)*
Poa	*Paniceae*	*Panicum bisulcatum*	*28(M)*
Poa	*Paniceae*	*Panicum miliaceum^a^*	−1.0±4.0(L)
Poa	*Paniceae*	*Janochloa antidotalis^a^*	−18(L)
Poa	*Paniceae*	*Urochloa texana^a^*	−2.0(L)
Poa	*Paniceae*	*Megathyrsus maximus^a^*	*37*±*34(M)*
Poa	*Paniceae*	*Digitaria sanguinalis^a^*	5.0(L)
Poa	*Paspaleae*	*Rugoloa hylaeica^a^*	*24(M)*
Poa	*Paspaleae*	*Rugoloa polygonata^a^*	−4.0(L)
Poa	*Paspaleae*	*Steinchisma hians* ^*b*^	10±34(L)
Poa	*Paspaleae*	*Paspalum dilatatum^a^*	0.0±6.0(L)
Poa	*Andropogoneae*	*Zea mays^a^*	1.0±5.0(L)
Poa	*Andropogoneae*	*Sorghum bicolor^a^*	6.0±5.0(L)
Ast	*Cichorieae*	*Lactuca sativa*	16(L)
Ast	*Cichorieae*	*Taraxacum officinale*	13(L)
Ast	*Heliantheae*	*Zinnia hybrida*	*38(M)*
Ast	*Heliantheae*	*Geraea canescens*	9.0(L)
Ast	*Heliantheae*	*Helianthus annuus*	*39(M)*
Ast	*Heliantheae*	*Xanthium strumarium*	0.0(L)
Ast	*Tageteae*	*Flaveria trinervia^a^*	−2.0(L)
Sol	*Petunieae*	*Petunia hvbrida*	*39(M)*
Sol	*Nicotianeae*	*Nicotiana rustica*	58(H)
Sol	*Nicotianeae*	*Nicotiana tabacum*	54±9(H)
Sol	*Capsiceae*	*Capsicum frutecens*	53(H)
Sol	*Physaleae*	*Physalis pruinosa*	*30(M)*
Sol	*Solaneae*	*Solanum dulcamara*	*39(M)*
Sol	*Solaneae*	*Solanum nigrum*	55(H)
Sol	*Solaneae*	*Solanum lycopersicum*	*29*±*4.0(M)*
Sol	*Solaneae*	*Solanum tuberosum*	47±8(H)
Sol	*Solaneae*	*Solanum melongena*	57(H)
Car	*N/A*	*Amaranthus retroflexus^a^*	−1.0(L)
Car	*N/A*	*Amaranthus tricolor*	5.0(L)
Car	*Beteae*	*Beta vulgaris*	46±19(H)
Car	*Anserineae*	*Spinacia oleracea*	0.0±10(L)
Car	*Chenopodieae*	*Chenopodium album*	−6.0±8.0(L)
Car	*Chenopodieae*	*Atriplex pentandra^a^*	1.0(L)
Car	*N/A*	*Mesembryanthemum crystallinum* ^*c*^	23(M)
Car	*N/A*	*Portulaca grandiflora^a^* ^*c*^	14±16(L)
Car	*N/A*	*Portulaca oleracea^a^* ^*c*^	−2.0(L)
Sax	*N/A*	*Crassula argentea* ^*c*^	45(H)
Sax	*N/A*	*Kalanchoe blossfeldiana* ^*c*^	−80(L)
Sax	*N/A*	*Kalanchoe daigremontiana* ^*c*^	49(H)
Sax	*Telephieae*	*Hylotelephium spectable* ^*c*^	−30(L)
Myr	*Onagreae*	*Camissonia brevipes*	0.0(L)
Cuc	*Cucurbiteae*	*Cucurbita pepo*	*41(M)*
Cuc	*Benincaseae*	*Cucumis sativus*	62±16(H)
Ros	*Ficeae*	*Ficus elastica*	54(H)
Fab	*Bauhinieae*	*Bauhinia galpinii*	*43(M)*
Fab	*Cassieae*	*Senna marilandica*	*36(M)*
Fab	*Mimosoideae*	*Neltuma julifora* ^*c*^	**89(VH)**
Fab	*Mimosoideae*	*Mimosa diplotricha*	63(H)
Fab	*Mimosoideae*	*Desmanthus illinoensis*	*37(M)*
Fab	*Mimosoideae*	*Desmanthus virgatus*	15(L)
Fab	*Amorpheae*	*Amorpha fruticosa*	*39*±*15(M)*
Fab	*Amorpheae*	*Dalea leporina*	45±2.0(H)
Fab	*Dalbergieae*	*Adesmia exilis*	60(H)
Fab	*Dalbergieae*	*Adesmia muricata*	56(H)
Fab	*Dalbergieae*	*Zornia braziliensis*	54(H)
Fab	*Dalbergieae*	*Aeschynomene indica*	50(H)
Fab	*Dalbergieae*	*Arachis hypogaea*	*23(M)*
Fab	*Dalbergieae*	*Stylosanthes hamata*	65(H)
Fab	*Sophoreae*	*Sophora alopecuroides*	*29(M)*
Fab	*Sophoreae*	*Sophora chrysophylla*	15(L)
Fab	*Sophoreae*	*Sophora sp.*	47(H)
Fab	*Crotalarieae*	*Crotalaria juncea*	*33(M)*
Fab	*Crotalarieae*	*Lotononis bainesii*	*32*±*32(M)*
Fab	*Genisteae*	*Lupinus albus*	0.0±0.0(L)
Fab	*Genisteae*	*Lupinus albicaulis*	*20(M)*
Fab	*Genisteae*	*Lupinus perennis*	10(L)
Fab	*Genisteae*	*Lupinus polyphyllus*	2.0(L)
Fab	*Genisteae*	*Lupinus sericeus*	17±16(L)
Fab	*Genisteae*	*Laburnum alpinum*	51±8.0(H)
Fab	*Genisteae*	*Genista tinctoria*	*25(M)*
Fab	*Robineae*	*Robinia pseudoacacia*	64(H)
Fab	*Sesbanieae*	*Sesbania sesban*	*39(M)*
Fab	*Loteae*	*Ornithopus compressus*	*35(M)*
Fab	*Loteae*	*Tetragonolobus purpureus*	50(H)
Fab	*Loteae*	*Coronilla scorpioides*	*27(M)*
Fab	*Loteae*	*Coronilla varia*	70(H)
Fab	*Loteae*	*Lotus caucasicus*	*23(M)*
Fab	*Loteae*	*Lotus corniculatus*	*43(M)*
Fab	*Glycyrrhizaea*	*Glycyrrhiza echinata*	54(H)
Fab	*Glycyrrhizaea*	*Glycyrrhiza sp*	16(L)
Fab	*Cicereae*	*Cicer arietinum*	1.0±1.0(L)
Fab	*Galegeae*	*Galega orientalis*	49(H)
Fab	*Vicieae*	*Vicia dasycarpa*	9.0(L)
Fab	*Vicieae*	*Vicia sativa*	0.0(L)
Fab	*Vicieae*	*Vicia hybrida*	7.0(L)
Fab	*Vicieae*	*Vicia faba*	8.0±12(L)
Fab	*Vicieae*	*Vicia ervilia*	3.0(L)
Fab	*Vicieae*	*Lens culinaris*	0.0±1.0(L)
Fab	*Vicieae*	*Lathyrus odoratus*	0.0(L)
Fab	*Vicieae*	*Lathyrus sativus*	1.0±1.0(L)
Fab	*Vicieae*	*Pisum fulvum*	0.0(L)
Fab	*Vicieae*	*Pisum elatius*	0.0±0.0(L)
Fab	*Vicieae*	*Pisum sativum*	2.0±7.0(L)
Fab	*Trifoliae*	*Ononis natrix*	*31(M)*
Fab	*Trifoliae*	*Ononis pubescens*	*34(M)*
Fab	*Trifoliae*	*Trifolium glomeratum*	*27*±*17(M)*
Fab	*Trifoliae*	*Trifolium repens*	*39*±*9.0(M)*
Fab	*Trifoliae*	*Trifolium pratense*	55±10(H)
Fab	*Trifoliae*	*Medicago sativa*	*29*±*12(M)*
Fab	*Trifoliae*	*Melilotus albus*	*46(M)*
Fab	*Trifoliae*	*Melilotus officinalis*	60(H)
Fab	*Trifoliae*	*Trigonella foenum-graecum*	*33*±*13(M)*
Fab	*Hedysareae*	*Hedysarum alpinum*	0.0(L)
Fab	*Hedysareae*	*Hedysarum coronarium*	4.0(L)
Fab	*Hedysareae*	*Onobrychis viciifolia*	0.0±0.0(L)
Fab	*Astragalaeae*	*Oxytropis lambertii*	10(L)
Fab	*Astragalaeae*	*Oxytropis pilosa*	*26(M)*
Fab	*Astragalaeae*	*Astragalus cicer*	62±25(H)
Fab	*Indigofereae*	*Indigofera sp.*	*29(M)*
Fab	*Indigofereae*	*Cyamopsis tetragonoloba*	*37(M)*
Fab	*Abreae*	*Abrus precatorius*	62(H)
Fab	*Millettieae*	*Tephrosia polystachya*	*43(M)*
Fab	*Millettieae*	*Tephrosia villosa*	52(H)
Fab	*Desmodieae*	*Lespedeza capitata*	75(H)
Fab	*Desmodieae*	*Lespedeza daurica*	*28(M)*
Fab	*Desmodieae*	*Desmodium adscendens*	56(H)
Fab	*Phaseoleae*	*Pueraria lobata*	*23(M)*
Fab	*Phaseoleae*	*Glycine max*	*41*±*16(M)*
Fab	*Phaseoleae*	*Vigna unguiculata*	56±10(H)
Fab	*Phaseoleae*	*Vigna acontifolia*	50(H)
Fab	*Phaseoleae*	*Vigna radiata*	55±34(H)
Fab	*Phaseoleae*	*Vigna mungo*	64(H)
Fab	*Phaseoleae*	*Phaseolus microcarpus*	**82(VH)**
Fab	*Phaseoleae*	*Phaseolus glabellus*	62(H)
Fab	*Phaseoleae*	*Phaseolus xanthotrichus*	74(H)
Fab	*Phaseoleae*	*Phaseolus grayanus*	50(H)
Fab	*Phaseoleae*	*Phaseolus angustissimus*	63(H)
Fab	*Phaseoleae*	*Phaseolus filiformis*	**88(VH)**
Fab	*Phaseoleae*	*Phaseolus vulgaris*	**86±9.0(VH)**
Fab	*Phaseoleae*	*Phaseolus acutifolius*	**80±7.0(VH)**
Fab	*Phaseoleae*	*Phaseolus coccineus*	70±14(H)
Fab	*Phaseoleae*	*Phaseolus leptostachyus*	**92(VH)**
Fab	*Phaseoleae*	*Phaseolus lunatus*	59±13(H)
Fab	*Phaseoleae*	*Phaseolus maculatus*	*39(M)*
Fab	*Phaseoleae*	*Macroptilium atropurpureum*	54(H)
Fab	*Psoraleeae*	*Psoralea cinerea*	*22(M)*
Fab	*Psoraleeae*	*Psoralea sp*	*38(M)*
Fab	*Psoraleeae*	*Psoralea tenax*	16(L)
Mal	*Clusieae*	*Clusia fluminensis* ^*c*^	**85(VH)**
Bra	*Arabidopsidea*	*Arabidopsis thaliana*	2.0±3.0(L)
Bra	*Camelinieae*	*Camelina sativa*	7(L)
Bra	*Brassiceae*	*Moricandia arvensis* ^*b*^	6.0(L)

Note: Dark inhibition values listed are species means with standard deviations included when species have multiple values. Levels of dark inhibition are Low (L, <18%, plain), Moderate (M, 18-44%, italicized), High (H, 44-77%, underlined), and Very High (VH, >77%, underlined and emboldened). Orders abbreviated: Nymphales (Nym), Alismatales (Ali), Asparagales (Asp), Poales (Poa), Asterales (Ast), Solanales (Sol), Caryophyllales (Car), Saxifragales (Sax), Myrtales (Myr), Cucerbitales (Cuc), Rosales (Ros), Fabales (Fab), Malphigeales (Mal), and Brassicales (Bra).

^*a*^C4 species.

^*b*^C2 Species.

^*c*^CAM Species.

### Data visualization

**Fig. 1.**
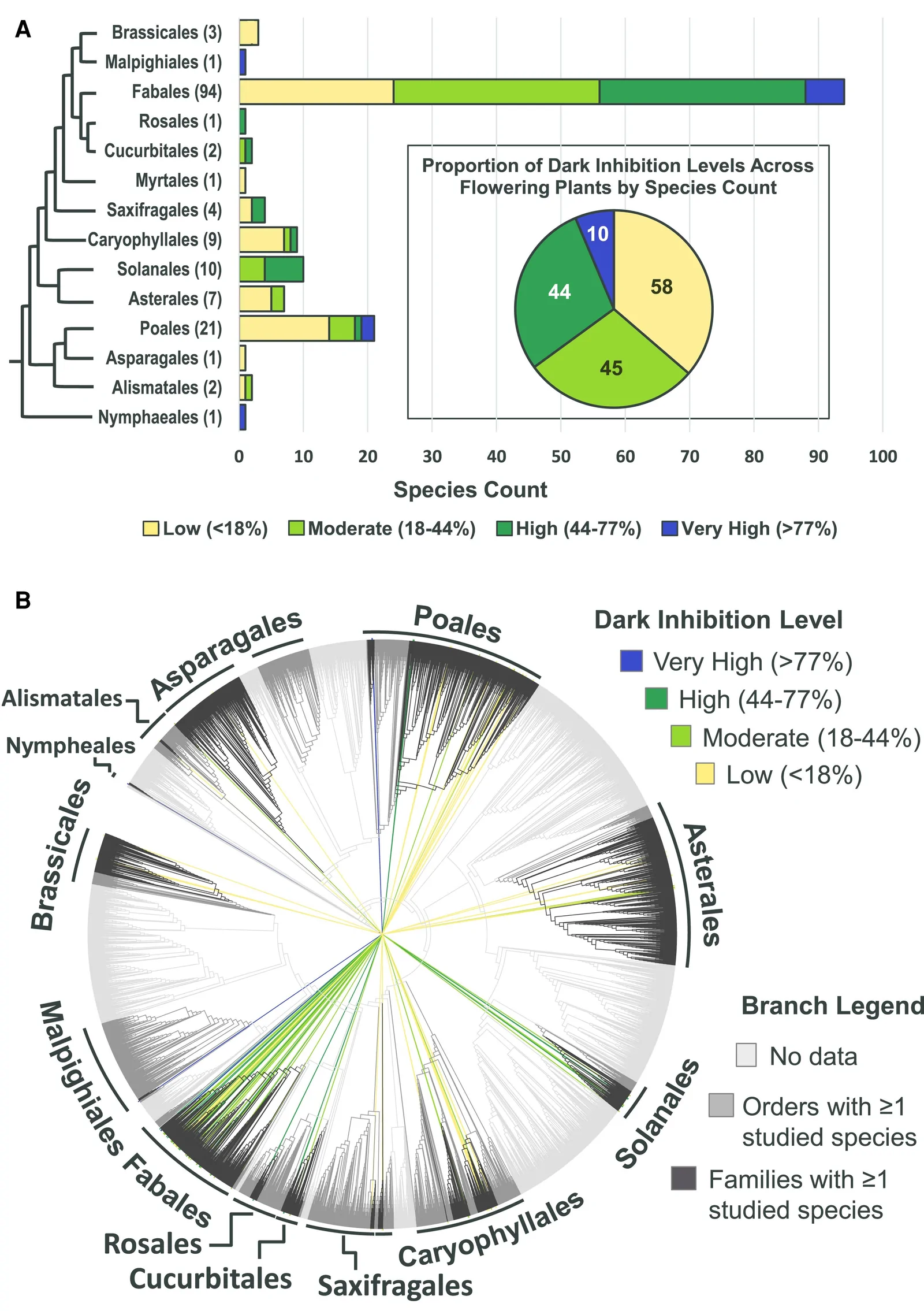
Distribution of Rubisco dark inhibition levels across flowering plant orders. (A) Simplified tree showing the phylogenetic relationship of 14 orders represented in the literature. Stacked bars represent the average species dark inhibition level, grouped from low, to moderate, high, and very high. In parentheses is the number of unique species studied within each order. Inset: breakdown of the proportion of dark inhibition levels across all species studied, numbered by count. (B) Phylogenetic tree of >10 000 flowering plant species coloured by dark inhibition levels at the genera level. Branches are coloured light grey for no species data available, grey for orders with data available for one or more species, and dark grey for families with data on dark inhibition available for one or more species. Lines radiating from the centre of the tree are connected to respective genera and coloured to indicate the average dark inhibition level of each genus. Dark inhibition levels are low (<18%), moderate (18–44%), high (44–77%), and very high (>77%).

**Fig. 2.**
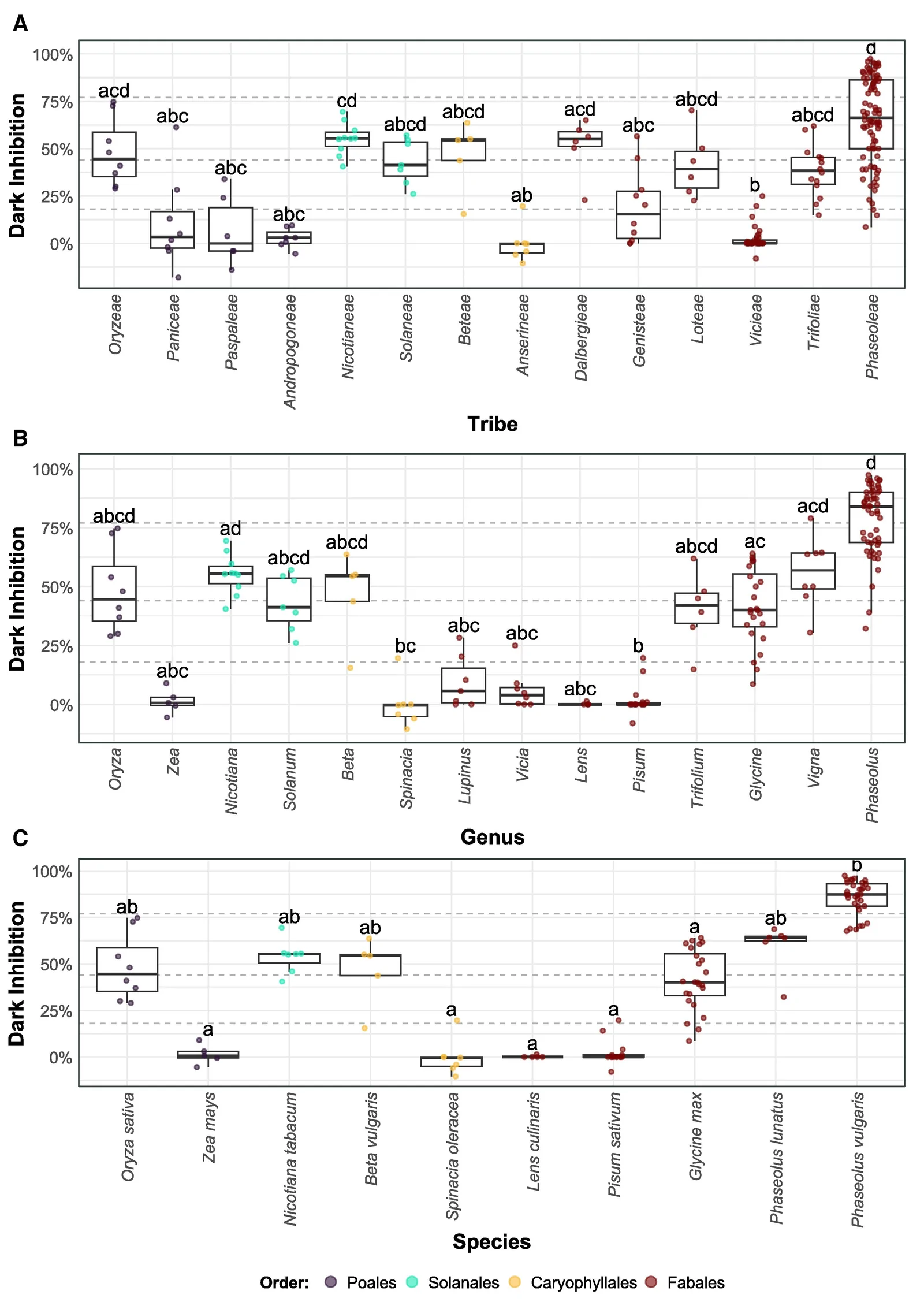
Variation in Rubisco dark inhibition distribution at different taxonomic levels. (A) Tribe, (B) genus, and (C) species. Only groups with a minimum of five data points are included. Data points grouped by order: Poales, Solanes, Caryophyllales, and Fabales. All groups are ordered by phylogenetic proximity. Dark inhibition ranges from 0 to 1, indicating 0% to 100% inhibition respectively. Box plots show medians and the first and third quartiles (25th and 75th percentiles), and whiskers extend from the hinge to the largest or smallest value. Kruskal–Wallis followed by Dunn post-hoc tests were performed to identify significant differences between groups, denoted by different letters (*P*<0.05).

Microsoft Excel was used to visualize data in Fig. 1A and Table 1. R was used to produce Fig. 2, using the following packages: ggplot2; multcompView; dplyr; readxl; agricolae; tibble; forcats; car; FSA; rcompanion; viridisLite; metafor; clubSandwich; robumeta; pracma; diptest; mclust; and multimode. The Kew Tree of life species tree was used as the backbone for creation of Fig. 1B , labelling and colouring branches and nodes using the Interactive Tree of Life website, and then further labelling the image in PowerPoint and Inkscape (Baker *et al*., 2022; Letunic and Bork, 2024). The coloured lines signifying average genera dark inhibition are for all monophyletic species in the tree of the given genera, even if only one species was studied for that genus. The tree can be accessed publicly here: (https://itol.embl.de/tree/148882475326681755591567). To see the tree with the same format as in Fig. 1B, collapse the tree nodes, and expand again by left clicking the central node. Then, in the coloured ranges section select ‘full’; this will show the coloured genera lines. Different branches may be collapsed for faster loading of the tree. All analyses and data are available in GitHub [cwnehls/NehlsRamos_etal_JxB_BriefComms_Code_and_Dataset; code for use in the paper, Nehls-Ramos et al. (2026). See readme for more information].

### Data analyses

Kruskal–Wallis and meta-regression modelling was used to assess whether data on Rubisco dark inhibition were broadly comparable between different Rubisco activity methods and methodological variations of these, as well as different publications. One-way ANOVA was not possible due to the consistent lack of a similar sample size between groups, and lack of normality as determined by the Shapiro–Wilk test. The full dataset for flowering plants obtained with studies employing the radiolabelled CO_2_ method was found to not be normally distributed and had unequal variance. Determination of Rubisco dark inhibition levels to establish groups of low to very high inhibition used a combination of McLust Gaussian mixture models, Hartigan dip test, followed by kernel density estimates to determine the number of modes in the data, and subsequently the position of modes and antimodes. For comparative analyses of dark inhibition across phylogenetic groups, Kruskal–Wallis followed by Dunn post-hoc tests, with Holm adjustment, were used due to the dataset not being normally distributed, and the sample sizes often being too small and showing uneven variance. All tests assumed a 0.05 significance level.

## Results

The dark inhibition data across studies carried a high sampling bias for the order Fabales, with far less sampling data regarding most other studied orders. Fabales, with 94 of the 157 species, represented ∼60% of the species-level data. Poales, with 21 species, was the only other order for which >10 species have had Rubisco dark inhibition data reported to date (Fig. 1A). This sampling bias is explained by two studies which contribute numerous species, all within the order Fabales (Holbrook *et al*., 1992; Sage, 1993). From the data available, we identified different levels of dark inhibition across species and provide a comprehensive survey on dark inhibition at species level, which presented interesting trends in distribution across flowering plants (Fig. 1; Table 1).

Proportional differences in species dark inhibition levels were visible across orders. Dark inhibition was classified into four levels, low (<18%), moderate (18–44%), high (44–77%), and very high (>77%) (see the Materials and methods). Focusing on the two most studied orders, Poales showed a greater proportion of low dark inhibition levels compared with the more even distribution across inhibition levels in Fabales (Fig. 1A). Grouping dark inhibition levels of species by phylogenetic order showed no clear trends across orders, though some had distinct trends even at low species count; for example, Solanales, with 10 species studied, had only moderate and high inhibition levels. Nonetheless, across all species studied, low inhibition was the most common level (57 of 157 species), being highly prevalent within Poales and Caryophyllales. Moderate and high levels were the next highest (45 species each). Very high inhibition levels were the rarest, with <7% of all species studied (10), half of these being members of Fabales.

To visualize further phylogenetic trends, the distribution of these levels of inhibition at the genus level was compared across a comprehensive phylogenetic tree for flowering plants. The categorized dark inhibition levels were overlaid across the Kew Tree of Life with branch tips at species level and labelling at genus level (Fig. 1B). The data available to date represent 14 of 64 orders of flowering plants, with wide coverage across the phylogeny tree. Within the gaps are smaller crop-relevant orders and families containing numerous spices, fruits, vegetables, and nuts. From current data, the more basal Poales branches containing the bromeliads and *Oryza* have higher dark inhibition compared with derived *Poaceae* grass lineages (Fig. 1B). Within Fabales, the highly studied *Fabaceae* family holds a wide spread of moderate and high inhibition genera, with low inhibition genera sequestered in distinct branches, while the *Phaseolus* branch trends towards very high inhibition (Fig. 1B).

Patterns at tribe level and below were explored using a comprehensive survey on dark inhibition at species level (Table 1). While nearly a quarter of Fabales species have low dark inhibition, these species are concentrated in the genera *Lupinus*, *Cicer*, *Hedysarum*, and *Onobrychis*, and the tribe *Viceae*. In contrast, other branches of Fabales, such as *Phaseolus* and *Vigna*, show above average Rubisco dark inhibition, with *Phaseolus* containing the most ‘very high’ level species of all studied genera in flowering plants (Table 1). Within this genus is *P. vulgaris*, the most studied species for dark inhibition. *Glycine max*, a close relative of *Phaseolus* and *Vigna*, had lower comparative mean inhibition and among the highest intraspecies variation (SD ±16%) for a well-studied species (Table 1; Fig. 2C). The driver for this surprising variation is discussed later. This variation in *G. max* partly reflects that as a major crop it has both a relatively high sample size and includes specific studies that identified large cultivar-level differences (Bowes *et al*., 1990; Holbrook *et al*., 1994), The 11 cultivars measured range from reported values of 8.6% to 64% (see full dataset).

Dark inhibition values showed inconsistent variation at both the genus and species level across flowering plants. Comparing groups with five or more observations, there were significant differences at tribe level, with six tribes showing significantly lower Rubisco dark inhibition levels compared with Phaseolae (Fig. 2A). At order level, significant differences were observed between the moderate and high inhibition Fabales and Solanales and the low inhibition Poales and Caryophyllales (Supplementary Fig. S3A). At genus level, this trend was also observed in relation to *Phaseolus* (Fig. 2B). This was observed despite the high range of variation in *Phaseolus*, with a median of 80% dark inhibition and an SD of >20%. There were eight genera with moderate to high inhibition, and in some cases significant differences were observed between these and the low inhibition genera *Pisum* and *Spinacia* (Fig. 2B). At the species level, significant differences were only identified for the low species versus the very high inhibition species, *P. vulgaris* (Fig. 2C). Some taxa, for example *Oryza sativa* and *Zea mays*, showed trends for lower and higher inhibition that were not significant, potentially due to low sample size resulting in insufficient statistical power. Conversely, for taxa with more data available, it was possible to identify clear and statistically significant differences. This was particularly the case for Fabales, with some genera showing near-zero dark inhibition compared with high levels observed in others, especially *Phaseolus* (Supplementary Fig. S3B).

## Discussion

Rubisco dark inhibition data published to date were compiled and analysed to identify trends that would help better understand the underlying role of this regulatory mechanism. Across flowering plants, only 157 species have been measured for Rubisco dark inhibition, representing <0.05% of the >300 000 known species, and only 14 of 64 orders (Baker *et al*., 2022; Antonelli *et al*., 2023). The data available show bias towards the Fabales order, consisting of nearly two-thirds of the total species measured with wide dark inhibition variation across species.

Intraspecies variation observed may potentially be due to biological variation, differences in sampling, plant age, growth conditions, assay methods, as well as a variable presence of daytime inhibitors (Vu *et al*., 1983; Moore *et al*., 1995). While factors such as assay methods and conditions were investigated here, for others there are insufficient data currently available. Cultivar differences were visible in *G. max*, even within the same study, though current data make robust comparisons of cultivar-level variation difficult (Holbrook *et al*., 1994). Species-level variance was present far less often in the well-studied bean *P. vulgaris* (Holbrook *et al*., 1994; Fig. 2C). *Glycine max* is tetraploid, having an additional ancestral genome duplication event compared with related beans (Yuan and Song, 2023). This higher dark inhibition variation may be explained in part by greater genotypic variation in production and regulation of CA1P. Chloroplast-level differences in Rubisco regulation by metabolites including CA1P binding affinity, and post-translational modifications probably also play a role (Parry *et al*., 2008; Amaral *et al*., 2024; Lobo *et al*., 2024). As the dark inhibition level was calculated by the difference of Rubisco activity in dark- and light-adapted samples, the presence of daytime inhibitors could mask the true level of dark inhibition (Keys *et al*., 1995; Orr *et al*., 2023). This appears likely in some species of the Poales, Caryophyllales, and particularly some in Saxifragales (Table 1; Supplementary Fig. S3A). Interestingly, despite Fabales containing species with almost no dark inhibition, very few of these species presented negative values, suggesting low daytime inhibitor accumulation in this order.

To facilitate more accurate and thorough understanding of dark inhibition will require additional data collection, potentially via streamlined methods such as linked 3PGA-NADPH assays; however, validation specifically for dark inhibition readings is needed (Sales *et al*., 2020). Additionally, further data for pre-dawn and midday Rubisco activity, if available, would be a valuable contribution to broaden our knowledge of dark inhibition. For accuracy in future data collection, the presence of daytime inhibitors should be accounted for. The use of sulfate to remove inhibitors from catalytic sites for maximal activity readings may serve as an effective control for activity measurements in the absence of inhibitors (Sage, 1993; Parry *et al*., 2008). Focusing on key phylogenetic clades of interest, such as with *Viceae* and its relatives, we may be closer to identifying the genetic controls for dark inhibition level variation.

For low inhibition species, trends in photosynthesis type were clearer than geographic trends. The majority of C_4_ species for which data are available had little to no dark inhibition, with the most inhibited species showing <40% dark inhibition (Table 1). C_2_ species and species with combined Crassulacean acid metabolism (CAM) and C_4_ photosynthesis types also had low inhibition (Table 1). Though data are limited, this trend suggests a potential correlation of low dark inhibition with C_4_-type photosynthetic adaptations in plants (Sage and Seemann, 1993). As the evolution of C_4_ photosynthesis often requires a careful balance of metabolites between cells, there may be stronger selection towards reducing and controlling the abundance of metabolites such as dark-synthesized inhibitors (Schlüter and Weber, 2020). While hot and dry environmental conditions are one of the recognized drivers for C_4_ photosynthesis, many low inhibition species are native outside of these conditions and are found across many environments (Stevens, 2025). Even with the somewhat limited data currently available, shifts towards low dark inhibition do not appear likely to be explainable by only climate differences. For phylogenetic trends, the low inhibition tribe *Viceae*, with Mediterranean origins, is closely related to the higher inhibition tribe *Trifoleae*, suggesting a nearby ancestral dark inhibition loss event (Table 1; Supplementary Fig. S3B; Holbrook *et al*., 1992; Schaefer *et al*., 2012).

In contrast to low inhibition, the low occurrence of ‘very high’ dark inhibition suggests potential specific niches for these species. This group included CAM species (*Ananas comosus*, *Bromelia pinguin*, *Neltuma julifora*, and *Clusia fluminensis*), an aquatic lily (*Nuphar lutea*), and several species in the genera *Phaseolus*, including common bean (*P. vulgaris*). No immediate common link between these species by morphology or lineage was obvious. Nearly all very high inhibition species are found in Central and South America; however, *N. lutea* is native to Eurasia (Stevens, 2025). An evolutionary pressure to retain higher levels of dark inhibition in tropical environments has been suggested previously in the literature, and this trend across otherwise disparate lineages would potentially support this (Holbrook *et al*., 1992). Given the high proportion of CAM species with very high dark inhibition—six of eight non-C_4_ CAM species having ‘high’ or greater dark inhibition levels—there may be a link between dark inhibition and CAM-type photosynthesis (Table 1). This photosynthesis type utilizes night-time stomatal opening, suggesting heightened Rubisco carbamylation, a condition which would favour increased CA1P binding and stronger Rubisco regulation (Holtum, 2023). Theoretically, the precursor 2-carboxy-D-arabinitol as well as malic acid should both build up in the vacuole in these plants diurnally (Moore *et al*., 1992), being readily available for CA1P synthesis at the onset of the night period.

Suggestions for the beneficial role of dark inhibition include the protection of Rubisco against proteolysis and reducing night-time Rubisco metabolite binding. However, neither of these theories appears to be supported by the large variation of inhibition observed, independent of species. While there is evidence that CA1P can have a protective role against proteolysis of Rubisco, and that variation of Rubisco–CA1P binding capacity may account for some of the dark inhibition variation (Khan *et al*., 1999; Parry *et al*., 2008, 2013), the results from this analysis do not directly support this first hypothesis. Species which are phylogenetically close would not be expected to have significant differences in Rubisco, such as *Viceae* versus *Trifoleae*; nonetheless, stark differences in dark inhibition are observed (Fig. 2A; Supplementary Fig. S2B). The *in vivo* regulation of Rubisco degradation and regulation across species is still not well understood; however, evidence suggests that this regulation by degradation in cereal grains neither is complex nor varies greatly across leaf age, unlike CA1P levels (Moore *et al*., 1995; Irving and Robinson, 2005; Feller *et al*., 2008; Buet *et al*., 2019). The absence of significant quantities of CA1P in many flowering plant species suggests a less essential role in plants. It is possible that CA1P could be an accessory byproduct for a non-essential ancestral metabolic pathway across plants (Moore and Seemann, 1992; Moore *et al*., 1992). Additionally, the role of CA1P is further confounded by the presence of its phosphatase, CA1Pase, which when overexpressed in wheat—a species without significant dark accumulation of CA1P—reduces Rubisco levels and carbon assimilation (Lobo *et al,* 2019).

The evolution of dark inhibition remains unclear even after 40 years of research, though some evidence suggests that dark inhibition may be more ancient than plants (Holbrook *et al*., 1992; MacIntyre *et al*., 1997). Given the presence of high inhibition in basal flowering plant branches and some evidence for dark inhibition in certain algae, dark inhibition probably did not arise in flowering plants but rather diversified within different lineages.

The analyses of Rubisco dark inhibition data available to date for flowering plants highlight both trends and conspicuous gaps in knowledge. Future analyses should consider factors that affect the chloroplast microenvironment where Rubisco resides, including geographical distribution, climate, canopy and light adaptations, plant and leaf morphology, Rubisco characteristics, and photosynthetic pathways. Identification of the *in vivo* impact of differences in dark inhibition, the core genes tied to dark inhibition regulation, and confirmation of CA1P as the main driver of dark inhibition will establish a stronger understanding of the physiological role of this enigmatic trait, with the potential to then impact crop productivity.

## Supplementary Material

erag090_Supplementary_Data

## Data Availability

The data collated and generated for this paper are included in the excel file found in https://github.com/cwnehls/NehlsRamos_etal_JxB_BriefComms_Code_and_Dataset. Within the GitHub file, the code used for analyses and figure generation are included. The angiosperm phylogeny tree is publicly available at https://itol.embl.de/tree/148882475326681755591567.

## Supplementary data

The following supplementary data are available at *JXB* online.

Fig. S1. Rubisco dark inhibition data by method and by publication.

Fig. S2. Determination of group levels for Rubisco dark inhibition.

Fig. S3. Rubisco dark inhibition for all plants with available data.

## Abbreviations

CA1P: 2-carboxy-D-arabinitol 1-phosphate
CAM: Crassulacean acid metabolism
3-PGA: 3-phosphoglyceric acid
