# Supplementary material for: Rubisco dark inhibition in angiosperms shows a complex distribution pattern
Source: J Exp Bot. 2026 Apr 15;77(10):2914–24. doi: 10.1093/jxb/erag090 (PMC13187672; doi:10.1093/jxb/erag090)
Supplement: erag090_Supplementary_Data [file erag090_supplementary_data.pdf]

Rubisco Dark Inhibition in Angiosperms Shows a Complex Distribution Pattern

Connor Nehls-Ramos, Elizabete Carmo-Silva, Douglas J. Orr*

Lancaster Environment Centre, Lancaster University, Lancaster, LA1 4YQ, UK

*Author for correspondence: d.j.orr@lancaster.ac.uk

Supplementary data:

Supplementary Fig. S1. Rubisco dark inhibition data by method and by publication.

Supplementary Fig. S2. Determination of group levels for Rubisco dark inhibition.

Supplementary Fig. S3. Rubisco dark inhibition for all plants with available data.

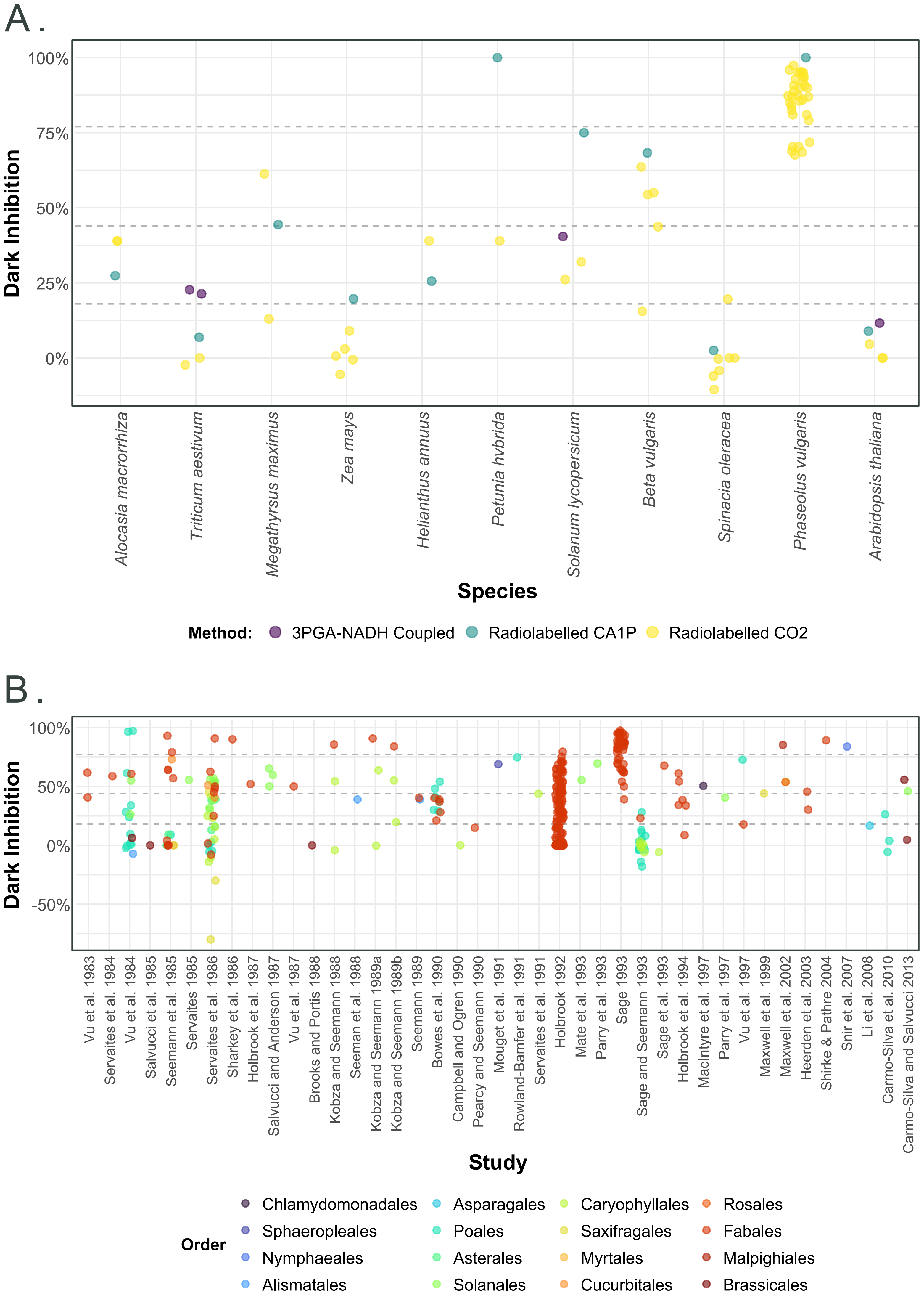
A.
100%
75%
50%
25%
0%
Dark Inhibition
Alocasia macrorrhiza
Triticum aestivum
Megathyrsus maximus
Zea mays
Helianthus annuus
Petunia hybrida
Solanum lycopersicum
Beta vulgaris
Spinacia oleracea
Phaseolus vulgaris
Arabidopsis thaliana
Species
Method:
3PGA-NADH Coupled
Radiolabelled CA1P
Radiolabelled CO2
B.
100%
50%
0%
-50%
Dark Inhibition
Vu et al. 1983
Servaites et al. 1984
Vu et al. 1984
Salvucci et al. 1985
Seemann et al. 1985
Servaites 1985
Servaites et al. 1986
Sharkey et al. 1986
Holbrook et al. 1987
Salvucci and Anderson 1987
Vu et al. 1987
Brooks and Portis 1988
Kobza and Seemann 1988
Seeman et al. 1988
Kobza and Seemann 1989a
Kobza and Seemann 1989b
Seemann 1989
Bowes et al. 1990
Campbell and Ogren 1990
Pearcy and Seemann 1990
Mouget et al. 1991
Rowland-Bamfer et al. 1991
Servaites et al. 1991
Holbrook 1992
Mate et al. 1993
Parry et al. 1993
Sage 1993
Sage and Seemann 1993
Sage et al. 1993
Holbrook et al. 1994
MacIntyre et al. 1997
Parry et al. 1997
Vu et al. 1997
Maxwell et al. 1999
Maxwell et al. 2002
Heerden et al. 2003
Shirke & Pathre 2004
Snir et al. 2007
Li et al. 2008
Carmo-Silva et al. 2010
Carmo-Silva and Salvucci 2013
Study
Order
Chlamydomonadales
Sphaeropleales
Nymphaeales
Alismatales
Asparagales
Poales
Asterales
Solanales
Caryophyllales
Saxifragales
Myrtales
Cucurbitales
Rosales
Fabales
Malpighiales
Brassicales

Supplementary Fig. S1. Variation of species Rubisco dark inhibition data by method and by publication. Dark inhibition was calculated as a ratio of Rubisco activity in the dark compared to the light. (A) Comparison of dark inhibition values by Rubisco activity method at species level. Species are ordered by phylogenetic relation. Methods are coloured yellow for radiolabelled CO2, teal for radiolabelled CA1P, and purple for 3PGA-NADPH coupled. (B) Comparison of total species data by publication with plant order denoted by the different colours. The x axis is not continuous and is ordered from oldest to newest by year from left to right.

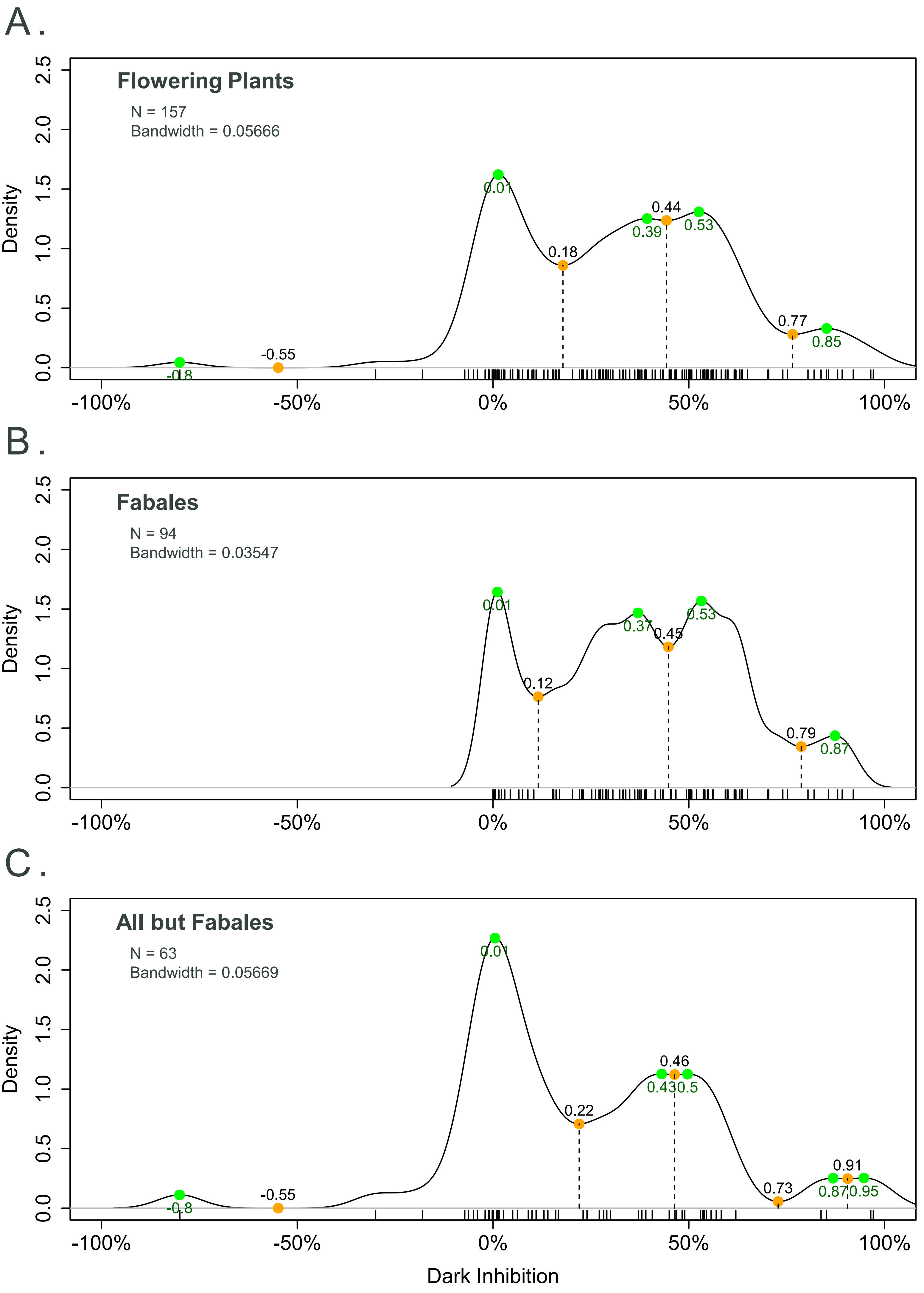
A.
Flowering Plants
N = 157
Bandwidth = 0.05666
0.01
0.18
0.39
0.44
0.53
0.77
0.85
-0.8
-0.55
B.
Fabales
N = 94
Bandwidth = 0.03547
0.01
0.12
0.37
0.45
0.53
0.79
0.87
C.
All but Fabales
N = 63
Bandwidth = 0.05669
0.01
0.22
0.46
0.43
0.5
0.73
0.91
0.87
0.95
-0.8
-0.55
Density
0.0
0.5
1.0
1.5
2.0
2.5
-100%
-50%
0%
50%
100%
Dark Inhibition

Supplementary Fig. S2. Determination of groupings for Rubisco dark inhibition levels. Kernel density estimates of average species dark inhibition datapoint distribution with modes and antimodes of significant peaks.
(A) All radiolabelled assay data for flowering plants,
(B) Fabales species only, or (C) all plants excluding Fabales. The peak height does not indicate number of species but the density of clustering of datapoints under the peak. The sample size (N) and bandwidth used to create the graph are as indicated.

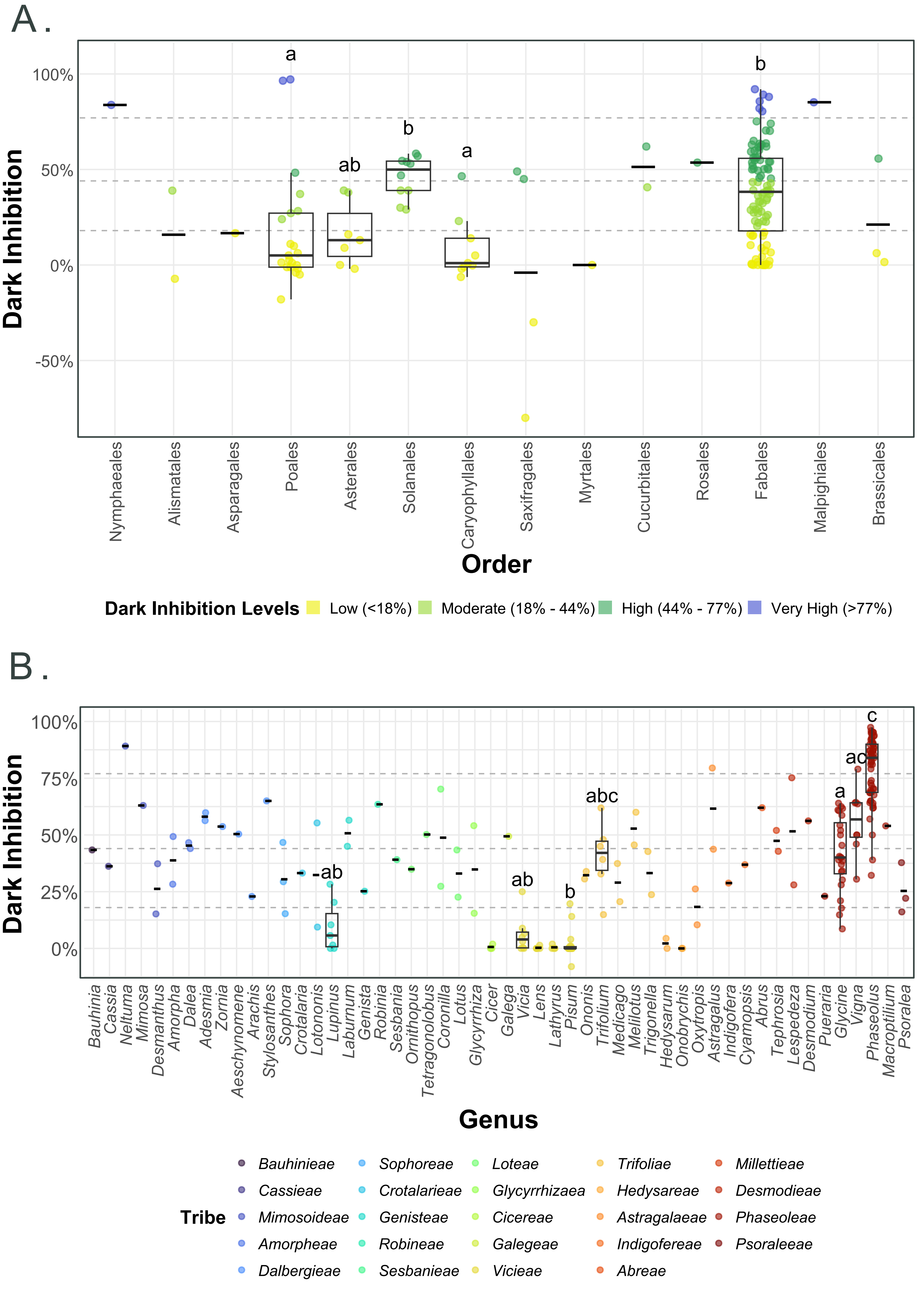

A.
100%
50%
0%
-50%
Dark Inhibition
a
ab
b
a
b
Nymphaeales
Alismatales
Asparagales
Poales
Asterales
Solanales
Caryophyllales
Saxifragales
Myrtales
Cucurbitales
Rosales
Fabales
Malpighiales
Brassicales
Order
Dark Inhibition Levels
Low (<18%)
Moderate (18% - 44%)
High (44% - 77%)
Very High (>77%)
B.
100%
75%
50%
25%
0%
Dark Inhibition
c
ac
a
abc
ab
ab
b
Bauhinia
Cassia
Neltuma
Mimosa
Desmanthus
Amorpha
Dalea
Adesmia
Zornia
Aeschynomene
Arachis
Stylosanthes
Sophora
Crotalaria
Lotononis
Lupinus
Laburnum
Genista
Robinia
Sesbania
Ornithopus
Tetragonolobus
Coronilla
Lotus
Glycyrrhiza
Cicer
Galega
Vicia
Lens
Lathyrus
Pisum
Ononis
Trifolium
Medicago
Melilotus
Trigonella
Hedysarum
Onobrychis
Oxytropis
Astragalus
Indigofera
Cyamopsis
Abrus
Tephrosia
Lespedeza
Desmodium
Pueraria
Glycine
Vigna
Phaseolus
Macroptilium
Psoralea
Genus
Tribe
Bauhinieae
Cassieae
Mimosoideae
Amorpheae
Dalbergieae
Sophoreae
Crotalarieae
Genisteae
Robineae
Sesbanieae
Loteae
Glycyrrhizaea
Cicereae
Galegeae
Vicieae
Trifoliae
Hedysareae
Astragalaeae
Indigofereae
Abreae
Millettieae
Desmodieae
Phaseoleae
Psoraleeae

Supplementary Fig. S3. Variation in Rubisco dark inhibition distribution at different taxonomic levels for all plants with available data. (A) All averaged species data by order coloured by dark inhibition level. (B) All species data group at genera level for Fabales coloured by tribe. Dark inhibition Dark inhibition was calculated as a ratio of Rubisco activity in the dark compared to the light. The x axes in both plots are ordered by phylogenetic proximity. For groups with less than 5 data points, the median line is shown. For groups with more than 5 data points, box plots show medians and the first and third quartiles (25th and 75th percentiles), and whiskers extend from the hinge to the largest or smallest value. Symbols represent individual species. Kruskal-Wallis followed by Dunn *post hoc* tests were performed to identify significant differences between groups, denoted by different letters (P<0.05).
